# Prospective Evaluation of Non-Compliant Severe Hemophilia Patients

**DOI:** 10.4274/tjh.galenos.2018.2018.0281

**Published:** 2019-05-03

**Authors:** Mehmet Can Uğur, Kaan Kavaklı

**Affiliations:** 1 University of Health Sciences, İzmir Bozyaka Training and Research Hospital, Clinic of Hematology, İzmir, Turkey; 2 Ege University Faculty of Medicine, Department of Pediatric Hematology, İzmir, Turkey

**Keywords:** Hemophilia, Compliance, Adolescent

## To the Editor,

Patient compliance with the determined treatment regimen is a current issue in the treatment of hemophilia, and there are many studies that report compliance issues in patients with hemophilia [1,2]. 

In our study, we applied a survey to 40 patients who participated in the adolescent workshop of Hemophilia Federation in March 2017, and we monitored 16 adolescent patients with severe hemophilia and investigated the changes in their compliance rates during a 1-year period. The survey was applied using face-to-face method. Subjects who were found to be non-compliant (patients who neglect to apply prophylaxis as recommended by their physicians) were monitored for 1 year. These subjects were reached by phone in months 6 and 12. The scope of these telephone calls was as follows: whether the subject was currently on prophylaxis, whether they were complying with the treatment plan, and the reasons for non-compliance.

This survey was an activity initiated for patients during a routine workshop. Therefore, we did not apply for ethics committee approval. 

There were a total of 40 subjects: thirty nine patients with severe hemophilia and 1 patient with von Willebrand disease (vWD). Among these subjects, 16 were found to be non-compliant: twelve patients with hemophilia A, 3 patients with hemophilia B, and 1 patient with vWD. The average age of these 16 subjects was 21.25 years. Ten patients (62.5%) were receiving prophylaxis. Two of the patients were middle school, 11 were high school, and 3 were university graduates.

There were 10 patients who were receiving prophylaxis at the start of study. The number of patients on prophylaxis increased to 12 and 14 at 6 and 12 months of follow-up. The rate of compliant patients was 43.75% in the sixth month and 56.25% in the first year.

It was determined that there were three reasons for non-compliance with the treatment: time constraints, being tired of the treatment, and problems with vascular access. The number of patients reporting these problems is presented in Table 1.

The definition of “acceptable compliance” can greatly different between studies. Generally, if patients administer at least 75% to 80% of the recommended doses, they are accepted to have perfect compliance [3]. Sixteen subjects who were found to be non-compliant were monitored for 1 year, and it was determined that the rate of compliance increased only to 56.25% in our prospective cohort study. 

Adolescent patients are more resistant to comply with recommended treatment plans. In this age group, the patients go through several biological, social, and emotional changes that influence their approach to the disorder [4]. Due to these factors, the non-compliance problem has a complicated nature that cannot be resolved through advising only. Treatment non-adherence is a chronic process in life-long chronic diseases such as hemophilia. As each patient is affected by different factors, it might be useful to conduct individual meetings with each patient instead of group trainings.

## Figures and Tables

**Table 1:**
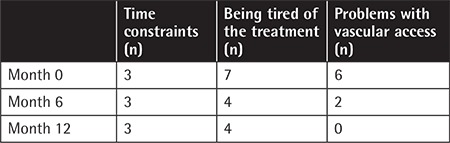
The causes of non-compliance with treatment of 16 patients.
